# Online Information on Painful Sexual Dysfunction in Women: Quality Analysis of Websites in SPANISH about Dyspareunia, Vaginismus and Vulvodynia

**DOI:** 10.3390/ijerph19031506

**Published:** 2022-01-28

**Authors:** Andrea Vicente-Neira, Virginia Prieto-Gómez, Beatriz Navarro-Brazález, Cristina Lirio-Romero, Javier Bailón-Cerezo, María Torres-Lacomba

**Affiliations:** 1 Physiotherapy in Women’s Health (FPSM) Research Group, Physiotherapy Department, Faculty of Medicine and Health Sciences, University of Alcalá, 28805 Madrid, Spain; andreavicente41@gmail.com (A.V.-N.); b.navarro@uah.es (B.N.-B.); cristina.lirio@uclm.es (C.L.-R.); bailonfisioterapia@gmail.com (J.B.-C.); maria.torres@uah.es (M.T.-L.); 2 Department of Nursery, Physiotherapy and Occupational Therapy, Faculty of Physiotherapy and Nursery, University of Castilla-La Mancha, 45071 Toledo, Spain; 3 Department of Physical Therapy, Centro Superior de Estudios Universitarios La Salle, Universidad Autónoma de Madrid, 28049 Madrid, Spain

**Keywords:** dyspareunia, vaginismus, vulvodynia, patient education, websites, health informatic, sexual dysfunction

## Abstract

The objective of this study was to evaluate the content, quality, and readability of websites containing information on dyspareunia, vaginismus, and vulvodynia in Spanish. Web pages were retrieved entering the terms “dyspareunia”, “vaginismus”, and “vulvodynia” in Google, Yahoo!, and Bing search engines. Two researchers employed the DISCERN and Bermúdez-Tamayo questionnaires to analyze the content and quality of the websites, and the INFLESZ scale to evaluate their readability. IBM SPSS^®^ version 25 statistical software was employed for data analysis. The internet search yielded 262 websites, 91 of which were included after applying the selection criteria. Websites with information on dyspareunia obtained median scores of 24 (30–21) in the DISCERN, 38 (41.0–35.5) in the Bermúdez-Tamayo, and 55.3 (57.2–50.9) in the INFLESZ tools. The results for websites on vaginismus revealed median scores of 23.5 (30–20) in the DISCERN, 37 (42–35) in the Bermúdez-Tamayo, and 52.9 (55.6–46.4) in the INFLESZ. Finally, the median scores for vulvodynia sites was 25.5 (30–20) in the DISCERN, 38 (43–33.7) in the Bermúdez-Tamayo, and 54.2 (57.3–47.2) in the INFLESZ. These outcomes indicate that the quality of information in these websites is very low, while the overall quality of the web pages is moderate. Sites on vaginismus and vulvodynia were “somewhat difficult” to read, while readability was “normal” for websites on dyspareunia. Healthcare professionals should be aware of the shortcomings of these websites and address them through therapeutic education with resources containing updated, quality information. This raises the need for health professionals to generate these resources themselves or for experts and/or scientific societies in the field to check the quality and timeliness of the contents, regardless of whether or not the websites are endorsed with quality seals.

## 1. Introduction

The World Health Organization describes sexual health as” a state of physical, emotional, mental and social well-being related to sexuality; it is not merely the absence of disease, dysfunction or infirmity. Sexual health requires a positive and respectful approach to sexuality and sexual relationships, as well as the possibility of having pleasurable and safe sexual experiences, free of coercion, discrimination, and violence. For sexual health to be attained and maintained, the sexual rights of all persons must be respected, protected, and fulfilled” [1]. Furthermore, the level of satisfaction derived from sexual activity in women depends on the correct synchronization and development of each phase of the sexual response, which can be conditioned by different biological, psychological, and socio-cultural factors, whose alteration can contribute to the development of female sexual dysfunctions (FSD) [2,3,4]. The Diagnostic and Statistical Manual of Mental Disorders-Fifth edition (DSM-5) of the American Psychiatric Association (APA) [5], which is one of the most widely used diagnostic tools at present, classifies FSD into three types of disorders: arousal or sexual interest, female orgasm, and genito-pelvic pain/penetration disorders (GPPPD). GPPPD include a variety of sexual disorders commonly associated with persistent pelvic pain, among which are usually found dyspareunia, vulvodynia, and vaginismus [2,3,4,5]. Dyspareunia is defined as genito-pelvic pain during or immediately after vaginal penetration. This condition affects 7.5% of sexually active women aged 16–74 years, with the highest prevalence reaching up to 10.4% and 9.5% in both more advanced (55–64 years) and younger (16–24 years) ages, respectively [6,7]. Superficial dyspareunia is closely related to vulvodynia, which is characterized by the presence of persistent pain in the vulvar area for more than three months without a clear identifiable cause [8]. Between 8 and 15% of women of reproductive age are estimated to present this dysfunction [9]. On the other hand, vaginismus affects 5–17% of women and can also be included within this group of GPPPD [10,11], where any form of vaginal penetration, whether sexual or not, is often painful or impossible [5]. Given the difficulty in differentiating and establishing criteria to discriminate between dyspareunia and vaginismus, since they respond to symptoms and/or health conditions that frequently coexist and are not mutually exclusive, the APA regrouped them within GPPPD in 2013 [5,12]. However, at present, these terms are often used indiscriminately, which generates confusion for both health professionals and patients.

Since pelvic pain and female sexual pain disorders are often associated with negative cognitive, emotional, behavioral, and sexual consequences, as well as with symptoms associated with urogynecological, instestinal, and sexual dysfunction, the scientific community and health professionals have suggested a biopsychosocial approach that addresses the different factors involved and responds to the individual needs of women [2,3,4]. This model considers the multidisciplinary action of physicians, physiotherapists, psychologists, nutritionists, and other health professionals, who, based on the existing alterations, improve or restore the affected functions during the practice of their competencies [2,3,4]. In this sense, therapeutic education is an indispensable, common, key element in the multidisciplinary approach to these dysfunctions, as it allows women to acquire specific knowledge and skills relevant for their disease and treatment, therefore enabling them to implement lines of action to improve, adapt, and preserve their sexual function [2,3,4,13]. In the women’s health realm, a lack of knowledge about pelvic floor dysfunction, including FSD, and treatment options is common among adult women. Only 18–50% of women with pelvic floor dysfunction are estimated to seek help from health care professionals [14,15].

The internet is a valuable and easily accessible source of information available to the citizen that, nevertheless, can be a double-edged sword: it allows patients to assume an active role in their recovery, but, on the other hand, the high volume of information is problematic when it comes to selecting quality information [16,17]. Additionally, psychosocial factors, such as fear, shame, and certain taboos associated with FSD, can potentially lead women to resort to the internet to seek information about FSD [18,19]. Disinformation in women’s sexual health can lead to developing erroneous cognitive schemes, such as extreme anxiety, feelings of guilt, unrealistic expectations, or fear of failure. Women with misguided sexual beliefs may develop negative thoughts that alter the way they experience a sexual encounter, increasing the likelihood to develop FSD [20,21]. Since misinformation can have a significant influence on the development and/or evolution of FSD [20,21], the objective of this study was to evaluate the content, quality, and readability of websites with information on dyspareunia, vaginismus, and vulvodynia in Spanish.

## 2. Materials and Methods

### 2.1. Study Design

A descriptive study was conducted to analyze the quality, content, and readability of web pages containing information on dyspareunia, vaginismus, and vulvodynia via the DISCERN questionnaire, Bermúdez-Tamayo questionnaire, and the INFLESZ scale.

### 2.2. Search Strategy

A comparison of the popularity of the terms dyspareunia, vaginismus, and vulvodynia was performed in Google trends (trends.google.es), applying the filters “Worldwide”, “Past 12 months”, “All categories”, and “Web search”. The browsers selected for the search were Google (Google.es), Yahoo! (es.yahoo.com), and Bing (bing.com), due to their popularity in “Statcounter” as the most used worldwide. All searches were performed in May of 2021.

Two researchers independently conducted an online search. Previously, the cache and history were cleared and the computer’s localization was deactivated. The terms dyspareunia, vaginismus, and vulvodynia were entered into each of the three search engines, and the first 20 websites were selected since users do not usually search for information beyond the first 20 hits.

An independent search of national registries of associations that had a website was also performed in June of 2021 to find patient associations linked to dyspareunia and/or vaginismus and/or vulvodynia in Spain and Latin America. No dyspareunia associations were identified, and the vaginismus and vulvodynia associations found (ANVAG and Associació Catalana de Vulvodínia) did not have a website.

### 2.3. Criteria for the Selection of Websites

All websites in Spanish containing information on dyspareunia and/or vaginismus and/or vulvodynia were included in this study.

The exclusion criteria were: duplicate websites or websites without a definition of dyspareunia and/or vaginismus and/or vulvodynia; offering advertising only; requiring subscription or payment to access the information; providing mostly PDF information, images, or videos; broken links and web content that redirected to other websites without the possibility of analyzing their readability. Websites were cataloged as: nonprofit; commercial websites marketing products or services; institutional, including government and professional organizations with medical qualification; free-of-charge information; and media owners.

### 2.4. Tools for Web Page Analysis

The DISCERN questionnaire is a valid and reliable tool used to evaluate the quality of written health information. It comprises 16 items grouped into three sections: items 1–8 to assess the reliability of the document, items 9–15 to evaluate the quality of treatment options, and a final section that assigns an overall rating to the quality of the document [22]. Each question is scored on a 5-point scale ranging from 5, indicating a clear “Yes”, to 1, indicating “No”. However, the intermediate scores 2–4 indicate that the document meets the criteria “partially”, and the decision of a higher or lower score depends on the subjective judgment of the evaluators. For this reason, based on the study by Cheneguin et al. [23], the two evaluators agreed on the rating of items based on the importance of the content, sources of information, date, additional support resources, description of how the treatment is performed with its benefits and risks, treatment options, and decision-making. The information quality was classified according to the obtained scores as: 16–29 = very low; 30–42 = low; 43–55 = moderate; 56–68 = good; and 69–80 = excellent [24].

The Bermúdez-Tamayo questionnaire is a valid and reliable instrument for evaluating the quality of health websites in Spanish. It includes 18 items divided into six sections: transparency and absence of conflict of interest, authorship, protection of personal data, updating of information, accountability, and accessibility. The overall score ranges from 17 to 54 and categorizes information quality as follows: 17–25 = very low; 26–33 = low; 34–40 = moderate; 41–47 = good; and 48–54 = excellent [25].

The INFLESZ scale, validated in Spanish and available online, qualifies the readability of a written text. The score is calculated using the Flesch-Szigriszt Index (IFSZ) available in the INFLESZ software (legible.es), which allows entering both written text and URLs. The software provides the score automatically, and the higher the score, the higher the readability of the text, with 100 being the highest possible. There are five levels: <40 = very difficult; 40–55 = somehow difficult; 55–65 = normal; 65–80 = fairly easy; and >80 = very easy [26].

### 2.5. Data Analysis

IBM SPSS^®^ version 25 statistical software was employed for data analysis. Quantitative variables were described by their median and interquartile range, after checking they did not fit a normal distribution using histogram graphs and the Shapiro-Wilk statistical test. Categorical variables were represented by their absolute and relative percentage frequencies.

The associations between quantitative dependent variables (score on each questionnaire) and polytomous qualitative independent variables (type of dysfunction) were measured with the Kruskal-Wallis test. Statistical significance was set at *p* < 0.05.

## 3. Results

### 3.1. Characteristics of the Included Websites

**Figure 1:**
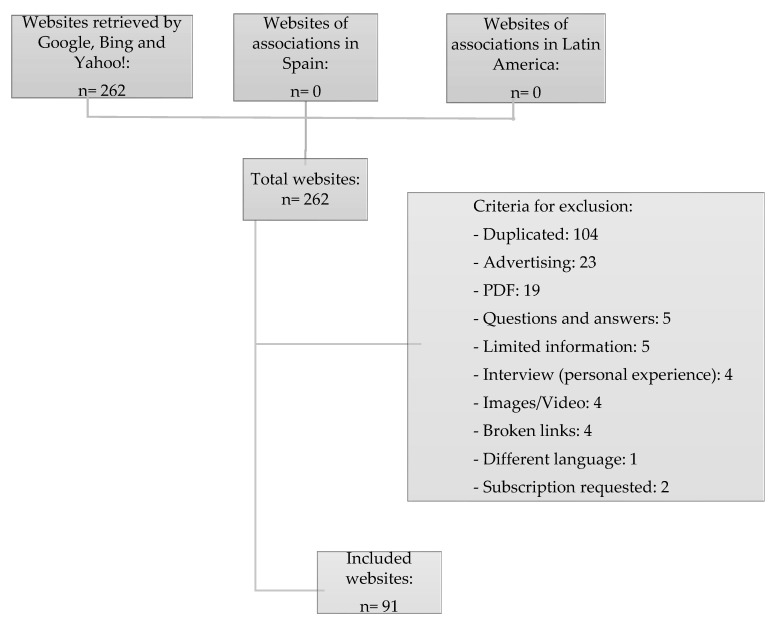
Selection process of websites.

Once the selection criteria were applied to the 262 websites found by the search engines, a total of 91 websites were included in the study (Figure 1). Table 1 shows the typology of the websites found for each of the terms entered.

### 3.2. Type of Information in Websites

#### 3.2.1. Dyspareunia

**Figure 2:**
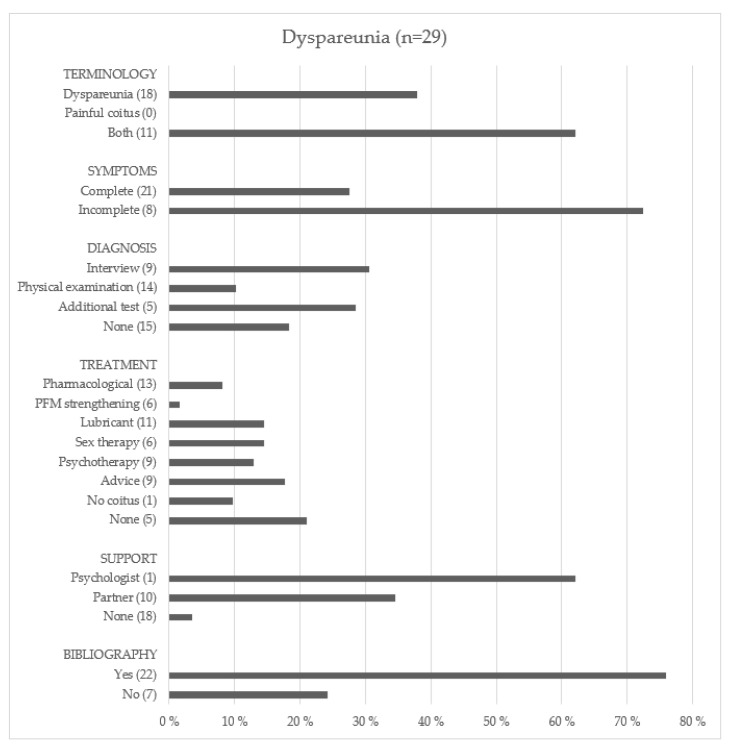
The type of information in websites on dyspareunia.

Figure 2 shows the type of information provided by the websites on dyspareunia. In terms of the terminology to refer to the condition of dyspareunia, eighteen of the websites (62.1%) termed it as such, and eleven (37.9%) of them, in turn, employed the term “painful coitus”. Websites also differentiated between superficial or deep dyspareunia, depending on the location. The symptoms mentioned were: vaginal pain that makes penetration difficult or impossible during intercourse (100%); burning sensation during sexual intercourse (15 websites: 51.7%); itching (6 websites: 20.7%); and anorgasmia (2 websites: 6.9%).

The diagnostic methods mentioned were: interview (9 websites: 31.0%), pelvic examination or gynecological examination (14 websites: 48.3%), and additional tests, such as urine analysis or biopsy (5 websites: 17.2%). However, fifteen of the examined websites (51.7%) did not mention any diagnostic procedure.

In terms of treatments, the most frequently recommended were: use of lubricant during sex (11 web pages: 37.9%), psychological therapy (9 web pages: 31.0%), advice, such as posture changes during sex or constant communication with the sexual partner (9 web pages: 31.0%), sex therapy (8 web pages: 27.6%), pelvic floor musculature strengthening exercises (6 web pages: 20.7%), and postponing intercourse until after recovery (1 web page: 3.4%). Five of the web pages analyzed did not mention any treatment. As for pharmacological treatment (referred in 13 web pages: 44.8%), the most frequently mentioned drugs were antibiotics (9 web pages: 31.0%), estrogen cream (8 web pages: 27.6%), and the medication ospemifene, that is used to alleviate menopausal changes (2 web pages: 6.9%). Some websites included tips to improve or avoid dyspareunia, such as effective communication with the partner (6 websites: 20.7%), changing posture during intercourse (7 websites: 24.1%), or spending more time on preliminary sexual acts (7 websites: 24.1%). Despite being a condition that affects couple relationships, eighteen websites (62.0%) did not emphasize the need for support for the women who suffer from dyspareunia, and only ten (34.5%) and one (3.4%) of the sites mentioned the importance of partner’s or psychological support, respectively. Finally, 76% of the web pages on dyspareunia cited relevant literature, while the remaining 24% did not mention sources of information.

#### 3.2.2. Vaginismus

**Figure 3:**
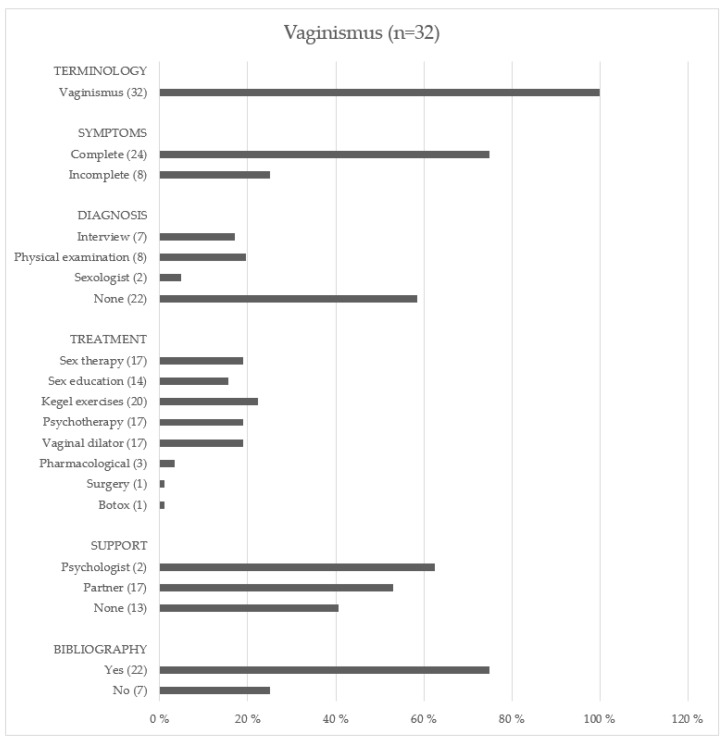
The type of information in websites on vaginismus.

Figure 3 shows the type of information provided by websites on vaginismus. “Vaginismus” was the only term used by the 32 websites (100%) selected. The symptoms were: pain upon penetration (29 web pages: 90.6%), difficulty (27 web pages: 84.4%) or impossibility (30 web pages: 93.7%) of vaginal penetration, involuntary contraction or spasm of the pelvic floor musculature (29 web pages: 90.6%) when intending or attempting penetration, burning sensation (8 web pages: 32.1%), anorgasmia (2 web pages: 6.2%), anxiety (2 web pages: 6.2%), and frustration (1 website: 3.1%).

In terms of diagnosis, most of the web pages did not mention any method (24 web pages: 75.0%), while others alluded to interviewing (7 web pages: 21.9%), pelvic or gynecological examination (8 web pages: 25.0%), and examination by a sexologist (2 web pages: 6.2%). The referred treatments were: Kegel exercises (20 web pages: 62.5%), sexual psychotherapy (17 web pages: 53.1%), use of vaginal dilators (17 web pages: 53.1%), sex education (14 web pages: 43.7%), and surgery and botulinum toxin (1 web page: 3.1%). Drug treatments were mentioned in three websites (9.4%), specifically antibiotics and muscle relaxant injections. Regarding the need for support for the person suffering from this condition, most websites stressed the importance of communication with the partner (17 web pages: 53.1%) or a psychologist (2 web pages: 6.2%), unlike the remaining 13 that did not mention emotional support (40.6%). Finally, bibliographic references were stated in 75.0% of websites on vaginismus.

#### 3.2.3. Vulvodynia

**Figure 4:**
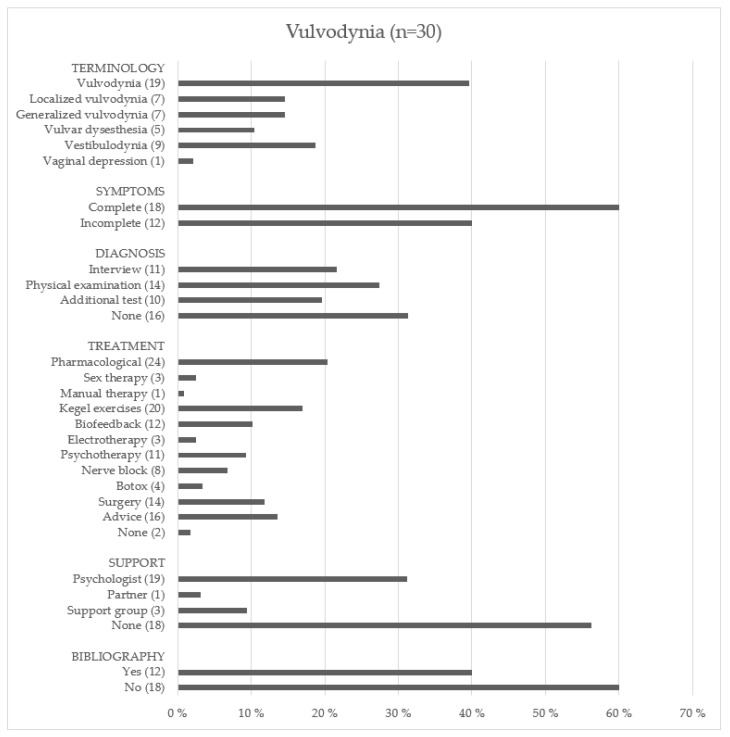
The type of information in websites on vulvodynia.

Figure 4 shows the information provided by the websites on vulvodynia. Regarding vulvar pain, the terminology used varied significantly from one website to another as follows: vulvodynia (19 websites: 63.3%), localized/generalized vulvodynia (7 websites: 23.3%), dysesthetic vulvodynia (5 websites: 16.6%), vestibulodynia or vulvar vestibulitis (9 websites: 30.0%), and vaginal depression (1 website: 3.3%). The symptoms described were: pain for at least 3 months that can be localized (18 web pages: 60.0%) or generalized (25 web pages: 83.3%), burning (28 web pages: 93.3%), dyspareunia (23 web pages: 76.6%), itching (14 web pages: 46.6%), and swelling or redness of the vulva (8 web pages: 26.6%). Forty percent of the web pages mentioned vulvar pain without a specific duration, so the symptoms were considered incomplete.

The most frequently cited pharmacological treatments were tricyclic antidepressants (21 web pages: 70.0%), anticonvulsants (18 web pages: 60.0%), and antihistamines (6 web pages: 20.0%). On the other hand, the use of topical medications, such as lidocaine ointment (17 web pages: 56.6%) or estrogen cream (14 web pages: 46.6%), was also mentioned. Other medical treatments included injections of interferon or corticosteroid (2 web pages: 6.6%) and nerve block (6 web pages: 20.0%). As for non-pharmacological treatments, Kegel exercises (20 web pages: 66.6%), pelvic floor musculature biofeedback (12 web pages: 40.0%), psychotherapy (11 web pages: 36.6%), and sex therapy (3 web pages: 10.0%) were found. In addition, a large number of web pages included tips to alleviate symptoms, such as using lubricant during sex (15 web pages: 50.0%), avoiding tight underwear (16 web pages: 53.3%), and not using perfumed hygiene products (14 web pages: 46.6%). Finally, literature references were present in only 40.0% of the included websites.

### 3.3. Quality of Health Information

Table 1 summarizes the results of the overall quality of the information contained in the websites evaluated with the DISCERN questionnaire. No statistically significant differences (*p* = 0.948) in the total score of this questionnaire were found between the three conditions:

Dyspareunia: a low median value of 24 (30–21) was obtained. Of the 29 sites, 20 (68.9%) scored between 16 and 29 points and were classified as “very low quality”, while the remaining nine (31.0%) scored between 30 and 42 points (low quality).

Vaginismus: the estimated median was low with a value of 23.5 (30–20). Of the total 32 included websites, 22 (68.7%) scored between 16 and 29 points (very low quality), nine (28.1%) scored between 30 and 42 (low quality), and only one website (3.1%) achieved scores between 43 and 55 (moderate quality).

Vulvodynia: the median score was low with 25.5 (30–20) points. Of the 30 websites, 21 (70.0%) were rated as “very low quality”, with a score between 16 and 29, and nine web pages (30.0%) as “low quality”, with scores of 30 to 42 points.

### 3.4. Quality of Websites

Table 2 shows the results of the quality of the websites, analyzed via the Bermúdez-Tamayo questionnaire. No statistically significant differences (*p* = 0.848) in the total score were observed between the three conditions:

Dyspareunia: the obtained median score of 38 (41–35.5) was moderate. Six (20.6%) websites scored between 26 and 33 (low quality), 16 (55.1%) websites between 34 and 40 (moderate quality), and seven (24.1%) between 42 and 47 (good quality).

Vaginismus: the outcomes showed a moderate median value of 37 (42–35). Seven (21.8%) web pages scored between 26 and 33 points (low quality), 14 (43.7%) obtained between 34 and 40 points (moderate quality), and 11 (34.3%) between 42 and 47 points (good quality).

Vulvodynia: the obtained median value of 38 (43–33.7) was moderate. Seven (23.3%) sites scored between 26 and 33 (low quality), 12 (40.0%) between 34 and 40 (moderate quality), and 11 (36.7%) between 42 and 47 points (good quality).

Information update obtained the lowest scoring for all three FSD conditions, with medians of 1 in all cases.

### 3.5. Readability of Websites

No statistically significant differences (*p* = 0.301) in the readability of websites were observed between the three conditions, as measured via the INFLESZ scale.

Dyspareunia: readability was classified as “normal” with a median of 55.3 (57.2–50.9). One (3.4%) site was classified as “very difficult” (<40), 14 (48.2%) as “somewhat difficult” (40–55), and 14 (48.2%) as “normal” (55–65) to read.

Vaginismus: readability was “somewhat difficult”, with a median of 52.9 (55.6–46.4). Two (6.2%) websites fell into the “very difficult” category (>40), 21 into the “somewhat difficult” category (40–55), and nine (28.1%) were considered “normal” to read (55–65).

Vulvodynia: readability was “somewhat difficult”, with a median of 54.2 (57.3–47.2). Two (6.6%) sites were classified as “very difficult” (>40), 17 (56.6%) as “somewhat difficult” (40–55), eight (26.6%) as “normal’ (55–65), and three (10.0%) as “fairly easy” (65–80) to read.

## 4. Discussion

To the authors’ knowledge, this is the first study to analyze the quality and readability of websites in Spanish on painful FSD. After analyzing all the selected web pages and interpreting the results, the present study found that the quality of websites in Spanish on dyspareunia, vaginismus, and vulvodynia retrieved by the main search engines was moderate. The quality of health information presented on the websites was classified as “very low” and readability was “somewhat difficult”, except for sites on dyspareunia where it was rated as “normal”. According to the literature consulted, the quality of health webpages varies from “moderate” to “unsatisfactory” and readability ranges from “normal” to “difficult” to read [23,27,28,29,30].

### 4.1. Websites Characteristics

Media owners was the most frequent typology of both dyspareunia and vaginismus websites, with percentages of 34.5% and 40.6%, respectively. However, most of the websites found on vulvodynia (46.6%) were institutional or professional. Conversely, the least frequent were non-profit websites or those that provide free-of-charge information. This may be because the media and institutions can invest part of their profits in the development and promotion of their website. Of note, the fields of psychology and physiotherapy were highly represented when searching for the terms vaginismus and vulvodynia, probably due to their professional aptitude in the recovery of these conditions [2,3,4].

### 4.2. Type of Information

Websites on dyspareunia, vaginismus, and vulvodynia mostly included information on symptoms and treatment, while diagnostic methods were not mentioned in over half of the websites found, a fact that is perceived as a shortcoming. In addition, the need to seek psychological support or to rely on one’s partner in the presence of these conditions was mentioned superficially.

In the case of dyspareunia, the most frequently mentioned treatment was pharmacological, which is selected according to the cause of the condition (e.g., menopause) [31,32]. Strengthening exercises of the pelvic floor musculature was the most frequently mentioned physiotherapeutic treatment, despite not being the only option for treating this disorder in this discipline [2,3,4,33,34]. Therapeutic education is an important pillar in the treatment of dyspareunia, although it was not mentioned in any of the retrieved websites [2,4,13]. On the other hand, psychotherapy or sex therapy appeared as a treatment option in only a few websites, although these therapies are often included in the multidisciplinary approach to painful FSD [2,3,4,5].

In terms of vaginismus, the most frequently mentioned symptoms (90.6%) were pain upon penetration, difficulty or impossibility of penetration, and involuntary contraction or spasm of the pelvic floor muscles. However, Reissing et al. emphasized that the latter symptom does not necessarily have to be present in all women, so it does not characterize the condition [35]. The most frequently mentioned treatments were Kegel exercises, sex education, sex therapy, and the use of vaginal dilators, although the information about them was scarce. There is evidence about the effectiveness of individual sex therapy, as well as couple sex therapy. On the other hand, cognitive behavioral therapy (CBT) is highly effective in women with mild to moderate vaginismus [36,37,38,39,40]. A study by Van Lankveld et al., in a sample of 117 women with primary vaginismus, compared the effectiveness of group CBT versus cognitive-behavioral bibliotherapy and a waiting-list control group. The CBT group obtained better outcomes as 21% of the women reported successful intercourse compared to the 15% in the bibliotherapy group [41]. Botulinum toxin was also mentioned in one of the web pages found, a method that is being used as a palliative treatment for pain with good results, although it has not been scientifically proven [41,42]. Several studies have shown the efficacy of different physical therapy approaches for treating vaginismus, especially pelvic floor strengthening exercises, although other options include therapeutic education, manual therapy, progressive desensitization, electrostimulation, or biofeedback [2,3,4,40,42].

The definition of vulvodynia proposed in a meeting of societies (the International Society for the Vulvovaginal Disease, the International Society for the Study of Women’s Sexual Health, and the International Pelvic Pain Society) in 2015 was “vulvar pain without a clear identifiable cause and with a duration of at least three months” [8]. However, 36.6% of the included websites mentioned pain without a specific duration of time. In terms of treatment, pharmacology is one of the major lines of action for treating pain, which was mentioned in 80% of the websites. Goldstein et al. do not recommend the use of corticosteroids given the limited evidence available and the relevant side effects [43]. Similarly, a review (2013) concluded the lack of scientific evidence available for the recommendation of anticonvulsants [44]. The approach to treating vulvodynia usually evolves from less invasive options, such as psychotherapy and physical therapy, to pharmacological treatment and even surgical intervention, if these alternatives fail, all of which were not discussed in the websites found [43,45].

### 4.3. Quality of Health Information

The quality of information on dyspareunia, vaginismus, and vulvodynia was “very low”. An article published in 2007 evaluated the quality of 101 websites on hypoactive sexual desire disorder in women [46]. Based on the method designed and described by Seidman et al. [47], they developed a tool to assess websites in terms of description of methods and validity, updating of information, and navigability. The results of that study indicated a low quality of the web pages on this type of sexual dysfunction, despite the use of a different procedure for the evaluation of the information [46]. Importantly, the low quality of the information in the websites could directly influence the development and prognosis of the health condition of affected women, in addition to hindering the acquisition of tools to improve their perceived self-efficacy and therapeutic adherence [13,21,48,49]. Health care professionals should be aware of the online information in Spanish-language websites that offer content on these conditions and explore their shortcomings, limitations, and potential incorrect or inaccurate information in order to recommend or warn patients about their reading, as well as to provide them with a contrast of quality information.

### 4.4. Quality of Websites

The updating of information was the questionnaire section that received the lowest scores in the 91 web pages examined. This is also true for other studies [50,51], such as that by Fernández Aranda et al. [52], where only 10 of the 61 websites analyzed described the process of the updating of information. This may stem from the difficulty to objectively evaluate this item since the degree of information updating varies, depending on the specific content being examined [25]. However, the current regulations for web pages require that the date of publication be mentioned, as well as the date of the utilized bibliographic references [25].

### 4.5. Readability

The readability score for pages about vaginismus and vulvodynia did not exceed 55 points, which is the limit at which a text is considered acceptable [26]. Therefore, the present study inferred that there is a “barrier” that prevents information on these painful FSD from reaching women. Alioshkin et al. analyzed the readability of fibromyalgia websites, including those retrieved by both search engines and from patient associations, and obtained average scores of 53.7 and 51.7 (somewhat difficult), respectively [23]. Castillo-Ortiz JD et al. reached similar conclusions when evaluating the readability of websites on rheumatoid arthritis: 9% of the websites were “very difficult”, 52% “somewhat difficult”, and 30% “normal” [28]. In contrast, dermatology websites seem to score better on the INFLESZ scale for readability, with a mean of 64.6 points (normal), which is the equivalent of an 8–10th school-level textbook [53].

### 4.6. Considerations to Improve Quality and Readability of FSD Websites

The results of the present study reveal the need to establish mechanisms to control the quality of the information on painful FSD in websites in Spanish, in addition to facilitating readability and comprehension, thus improving the accessibility to the information and avoiding the exclusion of women with low literacy skills. Obtaining a seal of quality could be one method for enhancing quality control for healthcare websites [54]. In this sense, given the heterogeneity in the criteria applied or the different accreditation processes followed by health websites that apply for quality seals, it would be very useful to unify the criteria established by the different international entities [54]. Furthermore, it is noteworthy that, although almost all the current quality seals guarantee the level of trust linked to the information content, none of them verifies the quality of the content, and the control of this content falls directly on the website “owners” [54]. In this regard, it would be of utmost importance that the entities responsible for awarding quality seals rely on experts and/or support from scientific societies in the field to verify and contrast the information provided, establish minimum update periods, and resort to tools, such as the INFLESZ scale [26], to ensure reaching acceptable levels of readability of the contents included. In this regard, it is highly advisable that the web pages include this type of services and that they periodically update the information as recommended by e-Europe and the American Medical Association [25], regardless of whether or not they have quality seals. Finally, it is worth noting that, ultimately, it is the responsibility of the health professional to use and/or recommend web content based on a prior analysis of the quality through the different tools available for this purpose, such as those used in this study.

### 4.7. Limitations

There are limitations to the present study. First, this study analyzed web pages on dyspareunia, vaginismus, and vulvodynia in Spanish, thus being of relevance to the 493 million Spanish speakers [55]. However, for a more complete analysis, the study could be extended to include websites in English.

Validated questionnaires, such as DISCERN and Bermúdez-Tamayo, which have been widely used for the evaluation of web pages, do not interpret the results with some of their items, depending on subjective assessment [22,25]. Specifically, DISCERN [22] does not clearly state which cases correspond to the intermediate scores, as is the case for the assignment of “Not applicable” and “Partially” in the Bermúdez-Tamayo questionnaire [25]. To overcome these limitations, two researchers conducted the analysis independently, having agreed and reviewed in advance those items that could give rise to confusion.

The DISCERN questionnaire is designed to analyze the quality of health information, more specifically that offered on treatment [22], while the Bermúdez-Tamayo questionnaire reviews the quality criteria of the websites so that the two complement each other [25]. However, neither of them evaluates the scientific veracity of the information offered, which is an important aspect when it comes to offering quality content to women who search the internet.

On the other hand, this is the first study that evaluated the quality and readability of websites on painful sexual dysfunctions, which precludes the comparison of findings. This fact confirms the need to improve the quality of health information available to patients, particularly in the field of women’s health, where a substantial lack of knowledge is observed.

## 5. Conclusions

The quality of information on websites about dyspareunia, vaginismus, and vulvodynia in Spanish is very low, while the overall quality of the pages is moderate. Sites on vaginismus and vulvodynia in Spanish were “somewhat difficult” to read, while readability was “normal” for those on dyspareunia.

In general, websites on painful FSD provide very limited information that is poorly contrasted with current scientific evidence. Health professionals must know the defects of these websites and overcome them via therapeutic education with updated quality information. This calls for health workers to generate such resources and for the websites to rely on experts and/or scientific communities that contrast the quality and updating of information, independently of whether or not the sites are backed by quality seals.

## Figures and Tables

**Table 1:** Distribution of scores obtained for information quality, overall and by health condition, according to the DISCERN questionnaire.

	Dyspareunia	Vaginismus	Vulvodynia
	Median (IQI)		
Readability of the publication	13 (19–11)	12 (17.5–11)	13.5 (19.25–10)
Quality of information on treatment options	9 (12.5–7)	9.5 (12–8)	9 (10.25–8)
Overall score	1 (1–1)	1 (1–1)	1 (1–1)
Total score	24 (30–21)	23.5 (30–20)	25.5 (30–20)

IQI: interquartile interval.

**Table 2:** Distribution of scores, overall and by categories, for website quality according to the Bermúdez-Tamayo questionnaire.

	Dyspareunia	Vaginismus	Vulvodynia
	Median (IQI)		
Transparence and absence of conflict of interests	12 (13–9)	12 (13.75–10.25)	12 (13–11)
Authorship	3 (4–3)	3 (4–2)	3 (6–2)
Personal data protection	3 (3–3)	3 (3–3)	3 (3–3)
Information updating	1 (1–1)	1 (1–1)	1 (1–1)
Responsibility	6 (6–5)	6 (6–5)	6 (6–4.75)
Accessibility	14 (16–14)	14 (16–11.5)	14 (15–12)
Total score	38 (41–35.5)	37 (42–35)	38 (43–33.75)

IQI: interquartile interval.

## Data Availability

Data are held securely by the research team and may be available upon reasonable request and with relevant approvals in place.

## References

[1] World Health Organization (2022). Sexual Health.

[2] Berghmans B. (2018). Physiotherapy for pelvic pain and female sexual dysfunction: An untapped resource. Int. Urogynecol. J..

[3] Al-Abbadey M., Liossi C., Curran N., Schoth D.E., Graham C.A. (2016). Treatment of female sexual pain disorders: A systematic review. J. Sex Marital. Ther..

[4] Engeler D., Baranowski A.P., Borovicka J., Dinis-Oliveira P., Elneil S., Hughes J., Messelink E.J., Williams A.C.d.C. (2018). EAU Guidelines on Chronic Pelvic Pain.

[5] American Psychiatric Association (2014). DSM-5. Manual Diagnóstico y Estadístico de los Trastornos Mentales.

[6] Mitchell K.R., Geary R., Graham C.A., Datta J., Wellings K., Sonnenberg P., Field N., Nunns D., Bancroft J., Jones K.G. (2017). Painful sex (dyspareunia) in women: Prevalence and associated factors in a British population probability survey. BJOG.

[7] Lee N.M.W., Jakes A.D., Llo Frodsham L.C.G. (2018). Dyspareunia. BMJ.

[8] Bornstein J., Goldstein A.T., Stockdale C.K., Bergeron S., Pukall C., Zolnoun D., Coady D., Bornstein J., Goldstein A., Zolnoun D. (2016). 2015 ISSVD, ISSWSH and IPPS Consensus Terminology and Classification of Persistent Vulvar Pain and Vulvodynia. Obstet. Gynecol..

[9] Reed B.D., Harlow S.D., Sen A., Legocki L.J., Edwards R.M., Arato N., Haefner H.K. (2012). Prevalence and demographic characteristics of vulvodynia in a population-based sample. Am. J. Obstet. Gynecol..

[10] Basson R., Althof S., Davis S., Fugl-Meyer K., Goldstein I., Leiblum S., Meston C., Rosen R., Wagner G. (2004). Summary of the recommendations on sexual dysfunctions in women. J. Sex. Med..

[11] Spector I.P., Carey M.P. (1990). Incidence and prevalence of the sexual dysfunctions: A critical review of the empirical literature. Arch. Sex. Behav..

[12] Lahaie M.-A., Amsel R., Khalifé S., Boyer S., Faaborg-Andersen M., Binik Y.M. (2014). Can Fear, Pain, and Muscle Tension Discriminate Vaginismus from Dyspareunia/Provoked Vestibulodynia? Implications for the New DSM-5 Diagnosis of Genito-Pelvic Pain/Penetration Disorder. Arch. Sex. Behav..

[13] Sánchez-Sánchez B., Arranz-Martín B., Navarro-Brazález B., Vergara-Pérez F., Bailón-Cerezo J., Torres-Lacomba M. (2021). How Do We Assess Patient Skills in a Competence-Based Program? Assessment of Patient Competences Using the Spanish Version of the Prolapse and Incontinence Knowledge Questionnaire and Real Practical Cases in Women with Pelvic Floor Disorders. Int. J. Environ. Res. Public Health.

[14] Mandimika C.L., Murk W., McPencow A.M., Lake A., Wedderburn T., Collier C.H., Connell K.A., Guess M.K. (2014). Knowledge of pelvic floor disorders in a population of community-dwelling women. Am. J. Obstet. Gynecol..

[15] Neels H., Wyndaele J.-J., Tjalma W.A.A., De Wachter S., Wyndaele M., Vermandel A. (2016). Knowledge of the pelvic floor in nulliparous women. J. Phys. Ther. Sci..

[16] McMullan M. (2006). Patients using the Internet to obtain health information: How this affects the patient-health professional rela-tionship. Patient. Educ. Couns..

[17] Tan S.S.-L., Goonawardene N. (2017). Internet Health Information Seeking and the Patient-Physician Relationship: A Systematic Review. J. Med. Internet Res..

[18] Kingsberg S.A., Schaffir J., Faught B., Pinkerton J.V., Parish S.J., Iglesia C.B., Gudeman J., Krop J., Simon J.A. (2019). Female Sexual Health: Barriers to Optimal Outcomes and a Roadmap for Improved Patient–Clinician Communications. J. Women’s Health.

[19] Feldhaus-Dahir M. (2009). Female sexual dysfunction: Barriers to treatment. Urol. Nurs..

[20] Ozmen H.E. (1999). Sexual myths and sexual dysfunctions. Psychiatr. World.

[21] Erbil N. (2019). Relationship between Sexual Myths and Sexual Function of Women. Int. J. Caring Sci..

[22] Charnock D., Shepperd S., Needham G., Gann R. (1999). DISCERN: An instrument for judging the quality of written consumer health information on treatment choices. J. Epidemiol. Community Health.

[23] Alioshkin-Cheneguin A., Salvat-Salvat I., Romay-Barrero H., Torres-Lacomba M. (2020). How good is online information on fibromyalgia? An analysis of quality and readability of websites on fibromyalgia in Spanish. BMJ Open.

[24] Charnock D. (1998). The DISCERN Handbook. Quality Criteria for Consumer Health Information on Treatment Choices.

[25] Bermudez-Tamayo C., Jimenez-Pernett J., Garcia Gutierrez J.F., Azpilicueta Cengotitobengoa I., Milena Silva-Castro M., Babio G., Castaño P. (2006). Questionnaire to evaluate health web sites according to European criteria. Aten Primaria.

[26] Barrio-Cantalejo I.M., Simón-Lorda P., Melguizo M., Escalona I., Marijuan M.I., Hernando P. (2008). Validación de la Escala INFLESZ para evaluar la legibilidad de los textos dirigidos a pacientes. Anales del Sistema Sanitario de Navarra.

[27] Kouhi A., Dabiri S., Mohseni A., Kouchakinezhad Eramsadati M. (2020). Evaluation of the Quality of Otolaryngology Information on Persian Websites. Iran. J. Otorhinolaryngol..

[28] Castillo-Ortiz J.D., Valdivia-Nuno J.J., Ramirez-Gomez A., Garagarza-Mariscal H., Gallegos-Rios C., Flores-Hernandez G., Hernandez-Sanchez L., Brambila-Barba V., Castaneda-Sanchez J.J., Barajas-Ochoa Z. (2017). Readability, relevance and quality of the information in Spanish on the Web for patients with rheumatoid arthritis. Reumatol. Clin..

[29] Schreuders E.H., Grobbee E.J., Kuipers E.J., Spaander M.C., Veldhuyzen van Zanten S.J. (2017). Variable Quality and Readability of Patient-oriented Websites on Colorectal Cancer Screening. Clin. Gastroenterol. Hepatol..

[30] Cisu T.I., Mingin G.C., Baskin L.S. (2019). An evaluation of the readability, quality, and accuracy of online health information regarding the treatment of hypospadias. J. Pediatr. Urol..

[31] Santoro N., Epperson C.N., Mathews S.B. (2015). Menopausal Symptoms and Their Management. Endocrinol. Metab. Clin..

[32] Vercellini P., Buggio L., Frattaruolo M.P., Borghi A., Dridi D., Somigliana E. (2018). Medical treatment of endometriosis-related pain. Best Pract. Res. Clin. Obstet. Gynaecol..

[33] Ghaderi F., Bastani P., Hajebrahimi S., Jafarabadi M.A., Berghmans B. (2019). Pelvic floor rehabilitation in the treatment of women with dyspareunia: A randomized controlled clinical trial. Int. Urogynecol. J..

[34] Wallace S.L., Miller L.D., Mishra K. (2019). Pelvic floor physical therapy in the treatment of pelvic floor dysfunction in women. Curr. Opin. Obstet. Gynecol..

[35] Reissing E.D., Binik Y.M., Khalifé S., Cohen D., Amsel R. (2004). Vaginal spasm, pain, and behavior: An empirical investigation of the diagnosis of vaginismus. Arch. Sex. Behav..

[36] Pacik P.T. (2014). Understanding and treating vaginismus: A multimodal approach. Int. Urogynecol. J..

[37] Negrin Perez M.C. (2010). Colegio Mexicano de Especialistas en Ginecologla y Obstetricia. Clinical practice guideline. Female sexual dysfunctions: Sexual pain disorders. Ginecol. Obstet. Mex..

[38] Bergeron S., Meana M., Yitzchak B., Khalife S., Levine S.B. (2010). Handbook of Clinical Sexuality for Mental Health Professionals.

[39] Fadul R., Garcia R., Zapata-Boluda R., Aranda-Pastor C., Brotto L., Parron-Carreno T., Alarcon-Rodriguez R. (2019). Psychosocial Correlates of Vaginismus Diagnosis: A Case-Control Study. J. Sex Marital Ther..

[40] Fugl-Meyer K.S., Bohm-Starke N., Damsted Petersen C., Fugl-Meyer A., Parish S., Giraldi A. (2013). Standard operating procedures for female genital sexual pain. J. Sex. Med..

[41] Van Lankveld J.J., ter Kuile M.M., de Groot H.E., Melles R., Nefs J., Zandbergen M. (2006). Cognitive-behavioral therapy for women with lifelong vaginismus: A randomized waiting-list controlled trial of efficacy. J. Consult. Clin. Psychol..

[42] Boyer S.C., Goldfinger C., Thibault-Gagnon S., Pukall C.F. (2011). Management of female sexual pain disorders. Adv. Psychosom. Med..

[43] Goldstein A.T., Pukall C.F., Brown C., Bergeron S., Stein A., Kellogg-Spadt S. (2016). Vulvodynia: Assessment and Treatment. J. Sex. Med..

[44] Leo R.J. (2013). A systematic review of the utility of anticonvulsant pharmacotherapy in the treatment of vulvodynia pain. J. Sex. Med..

[45] Rosen N.O., Dawson S.J., Brooks M., Kellogg-Spadt S. (2019). Treatment of Vulvodynia: Pharmacological and Non-Pharmacological Approaches. Drugs.

[46] Touchet B.K., Warnock J.K., Yates W.R., Wilkins K.M. (2007). Evaluating the quality of websites offering information on female hypoactive sexual desire disorder. J. Sex Marital Ther..

[47] Seidman J.J., Steinwachs D., Rubin H.R. (2003). Design and testing of a tool for evaluating the quality of diabetes consumer-information Web sites. J. Med. Internet Res..

[48] Beatriz-Navarro B., Vergara-Pérez F., Prieto-Gómez V., Sánchez-Sánchez B., Yuste-Sánchez Y.S., Torres-Lacomba M. (2021). What influences women to adhere to pelvic floor exercises after 2 physiotherapy treatment? A qualitative study for individualized pelvic health care. J. Pers. Med..

[49] Torres-Lacomba M., Navarro-Brazález B., Yuste-Sánchez M.J., Sánchez-Sánchez B., Prieto-Gómez V., Vergara-Pérez F. (2022). Women´s experiences on compliance of pelvic floor home exercises therapy and lifestyles changes for pelvic organ prolapse symptoms: A qualitative study. J. Pers. Med..

[50] De la Torre Barbero M.J., Estepa Luna M.J., Lopez-Pardo Martinez M., Leon Marquez M., Sanchez Laguna F., Toledano Redondo S. (2014). Evaluation of the Andalusia Public Health System hospital websites in the period 2010–2012. Rev. Calid. Asist..

[51] Hernandez-Morante J.J., Jimenez-Rodriguez D., Canavate R., Conesa-Fuentes M.C. (2015). Analysis of Information Content and General Quality of Obesity and Eating Disorders Websites. Nutr. Hosp..

[52] Fernández-Aranda M.I. (2016). Evaluación de calidad de páginas web sobre obstetricia y ginecología para las gestantes de atención primaria. Matronas Prof..

[53] Mazmudar R.S., Sheth A., Tripathi R., Scott J.F. (2021). Readability of online Spanish patient education materials in dermatology. Arch. Dermatol. Res..

[54] Padilla-Garrido N., Aguado-Correa F., Huelva-López L., Ortega-Moreno M. (2016). Comparative analysis of quality labels of health websites. Rev. Calid. Asist..

[55] Fernández-Vítores D. (2021). El Español: Una Lengua Viva.

